# Fabrication and characterization of different PbO borate glass systems as radiation-shielding containers

**DOI:** 10.1038/s41598-024-52071-x

**Published:** 2024-02-01

**Authors:** E. M. Abou Hussein, A. M. Madbouly

**Affiliations:** 1 Radiation Chemistry Department, National Center for Radiation Research and Technology (NCRRT), Egyptian Atomic Energy Authority (EAEA), Cairo, Egypt; 2 Radiation Safety Department, Nuclear and Radiological Safety Research Center (NRSRC), Egyptian Atomic Energy Authority, Cairo, Egypt

**Keywords:** Computational biophysics, Materials for optics, Environmental sciences, Risk factors

## Abstract

Three borate glasses of 50, 35, and 15 mol% PbO-doped Ce, Sb, or Mn ions were fabricated via the melting-annealing procedure. Their structural features were inspected before and after 250 kGy of gamma irradiation using FTIR and ESR techniques. The spectra of the ESR and FTIR vibrational bands remain constant, with a minor reduction in N4 and an enhancement in density values after irradiation, indicating the large structural stability and glass compactness. Many radiation shielding parameters were studied, such as gamma dose rate (µSv/h), dose transmission %, lifetime cancer risk %, macroscopic effective removal cross-section (∑_R_), mass stopping power, and projected range values ​​were considered for protons particles by SRIM Monte Carlo simulation code and ESTAR program. The whole data reveals the high radiation shielding efficiency of the glasses compared to other standard shields to be used as glass immobilizers for radioactive wastes or storage containers, e.g., for nuclear medicine units in hospitals.

## Introduction

Although nuclear power has great importance in many industrial, medical, and environmental fields, its advanced generation and the convoyed nuclear fuel cycle yield a variety of radioactive wastes that have become increasingly hazardous. Due to the radioactive contamination and harmful effects of exterior exposure to radiation, handling radioactive wastes is a very hazardous procedure^1^. So, it is mandatory to follow some strict radiation protection rules, such as treating and conditioning nuclear waste containers in a way that keeps dose rates below certain limits. For example, in shielding nuclear medical facilities, this process can be done by covering radioactive materials in containers to minimize radiation contact with medical employees during radiation therapy. Transporting and storing radioactive materials also require safe separation, which includes the preventing of  radiation waste leakages^2^.

Several safety and regulatory requirements must be met when disposing radioactive wastes, but it is a very expensive and difficult procedure because many activities must be performed in order to reduce the cost as much as possible^1^.

One of the most proficient and extremely desirable methods to deal with the problem of radioactive wastes is the fabrication of lightweight materials with radiation shielding efficiency, e.g., glass^1–3^. In recent years, glass has emerged as one of the most hopeful light materials to immobilize high-level radioactive wastes (HLWs) due to its complex structure. It has a stretchy structure for incorporating many types of waste elements by extensively atomic-bonding among the glassy network and radioactive wastes^4–6^. Borosilicate glasses, e.g., Pamela, SON68, and WAK^7^, are the most common glasses used as nuclear waste immobilizers.

The bezel proficiency of nuclear waste glass can be detected by its affinity to struggle with the released photons of ionizing radiation and stop their paths^6^. Subsequently, the chosen glass should have many properties, such as high structural stability against high radiation doses, mechanical steadfastness, thermal stability, and chemical durability in various leaching media^6,8^. The capability of a precise glass to inhibit radiation photons is controlled predominantly by its main host composition. The amorphous nature of the glassy structure assists in the housing of various metal ions, which afford the glass radiation shielding proficiency. So, the selection of each metal oxide and its concentration in the glassy network should be sensibly considered. Particularly, heavy metal ions afford the exceptional radiation shielding characteristics of their host glasses owing to their astounding mass attenuation coefficients related to their large atomic weights^9^. For instance, lead and bismuth oxides are the most common oxides used for developing radiation shielding properties in glass due to their heavy masses, small field strength, and high polarizability to capture radiation photons, e.g. Pb is the central component of radiation protection concrete. B_2_O_3_ glasses are also distinguishable in their properties due to their low cost, the simple way of preparation and shaping, and the tendency of boron to absorb radiation photons. The trigonal BO_3_ and tetrahedral BO_4_ are the central structural units in borate glasses. The accommodation of modifying oxides in the glassy network tends to transform BO_3_ to BO_4_ units by forming more non-bridging oxygens (NBO)^10^. While the incorporation of intermediate oxides like Pb^2+^ ions propose significant variations in the glass network correlated to its native environment and coordination’s by forming the expected resilient building units such as PbO_3_, PbO_4_, and PbO_6_^11^, Lead-borate glasses can be broadly used for radiation protection purposes for observers and workers in healthcare, industrial environments, veterinary medicine, and dentistry^9,12,13^. Furthermore, rare earth ions and transition metal ions enhance the radiation protection proficiency of their host glasses^14–16^ because of their ability to change their coordination to deal with radiation photons and heal the expected defect centers^17–20^.

For instance, Ce ions have highly required features correlated to their nature as rare earth ions having an outer surface electronic configuration of 5s^2^, 5p^6^, and 4f. orbitals^9^. Cerium ions can present as Ce^3+^ ↔ Ce^4+^ states in an equilibrium reaction to balance the negative charges on the neighboring tetrahedrons, helping in the formation of more connected bonds and relaxed or rigid glassy network. On the other side, Sb^3+^ has a massive weight and can form stable SbO_3_ trigonal pyramids in borate network, giving a compacted glassy network. Moreover, Mn^2+^ ions can exist in many oxidation states and participate as octahedral and/or tetrahedral units in the glassy network. Such induced ions are able to alter their valence states to absorb defect centers that may be caused by the effect of irradiation and inhibit the passage of radiation photons^9,21^.

Numerous examinations should be conducted on each selected glass composition to evaluate its tendency to inhibit the activity of ionizing radiation photons to its minimum level, such as structural stability, formation of free radicals because of breaking bonds, density, chemical durability, and many other experimental and theoretical radiation shielding parameters, so that the glass's capability to contain different radioactive wastes can be evaluated.

Further work was published indicating the promising radiation shielding application of the investigated glasses^21^. The objective of the present work is to continue the work on the prepared lead borate glasses and make a comparison test on the effect of 250 kGy of gamma radiation on some of their structural and physical properties using FTIR and ESR techniques to estimate their possible usage as storage containers for radioactive wastes. Additionally, Phy-X/PSD software was used to determine the macroscopic effective range cross-section of the fast neutron, ∑_R_, and the simulation code MCNP5 has also been inspected, e.g., gamma dose rate (µSv/h) from exposure to a radioactive source, dose transmission %, lifetime cancer risk % compared to other standard shields, and macroscopic effective removal cross-section (∑_R_). In addition, the projected range (Π_P_) and mass stopping power (ψ_p_) of protons (H ions) were estimated using the SRIM Monte Carlo simulation system and its subroutine TRIM. The continuous slowing down approximation (CSDA) range and the electron mass stopping power (ψ_e_) were both calculated using the ESTAR program. Then the best glass system for radiation shielding ability can be determined as promising storage containers for radioactive wastes.

## Materials and methods

### Preparation of glass systems

Three systems of different lead-borate glass compositions were organized by the communal melting-annealing method. The compositions of the fabricated glasses are scheduled in Table 1, and chemicals from Sigma-Aldrich Company (purity 99.9%) were used to prepare the glasses: H_3_BO_3_, PbO, K_2_CO_3_, Na_2_CO_3_, Al_2_O_3_, CeO_2_, Sb_2_O_3_, and MnO_2_. According to each glass composition listed in Table 1, three batches were exactly weighed using a sensitive balance (four digits ± 0.0001). The batches were differentiated carefully and ground into sufficient powders to be melted in porcelain crucibles in an electric muffle furnace with a temperature range of 950–1100 °C for 90–130 min. To achieve homogeneity, the crucibles were carefully stirred at 30-min intervals. After the complete melting, the homogenized melts were cast on heated stainless-steel molds and immediately transmitted to an annealing furnace at a temperature range of 350–400 °C to exclude stress or thermal strain excesses in the prepared samples. Then the annealing furnace was turned off after 1 h, leaving samples inside until the gradual cooling to room temperature with a rate of 30 °C.h^−1^. After that, bulk glass samples with a thickness = 2.98 ± 0.03 mm, or powered samples, were intended for characterization processes.

**Table 1:** Compositions and melting temperatures of the prepared lead borate glasses and their density values before and after gamma irradiation.

Glass Sample	Composition mol. %								Melting Temperature	Density Before irradiation g/cm^3^	Density after 250 kGy g/cm^3^
	B_2_O_3_	PbO	K_2_O	Na_2_O	Al_2_O_3_	CeO_2_	Sb_2_O_3_	MnO_2_			
G1	50	50				4			950 °C	5.26	5.30
G2	40	35	15				10		1100 °C	4.26	4.26
G3	60	15		15	5			5	1100 °C	3.34	3.38

### Characterization techniques

The FTIR absorption spectra of the prepared glasses were inspected before and after exposing them to 250 kGy of gamma radiation in a wavenumber range of 400–4000 cm^−1^ using the VERTEX 70, FT/ IR-430 spectrometer type at room temperature. N4 values were obtained from FTIR spectra according to the following relation: N_4_ (FTIR) = BO_4_/(BO_3_ + BO_4_) depending on the area under peaks that are associated with the corresponding sections of BO_3_ and BO_4_ units. Deconvolution of FTIR spectra was carried out using the Peak Fit program in Gaussian Amp. Mode.

The Bruker EMX spectrometer (X-band, Germany) was used to perform ESR measurements with the following operating parameters: microwave power of 2 mW, modulation amplitude of 3 G, sweep width of 200 G, microwave frequency of 9.72 GHz, time constant of 81.92 ms, and sweep time of 20.48 s at room temperature of 25 ± 2 °C. The ESR measurement was completed before and after exposing glasses to 250 kGy of gamma radiation.

Density (ρ) of the glasses was calculated at room temperature using the suspended weight method based on Archimedes principle^13,18^ according to the following relation:

1 $$\rho \, = \,\left\{ {a\backslash \left( {a\, - \,b} \right)} \right\}\, \times \,0.{86}\;g/cm^{{3}} ,$$

where, (ρ) is density of the glass sample, (a) and (b) are weights of the glass specimens in air and xylene, respectively and (0.86) is xylene density at 20 °C.

Irradiation was performed with a ^60^Co gamma cell (2000 Ci) with a dose rate = 0. 717 kGy/h at 25 ± 5 °C. The samples were placed in the gamma cell in such a way that each sample was exposed to the required identical dose.

**Figure 1:**
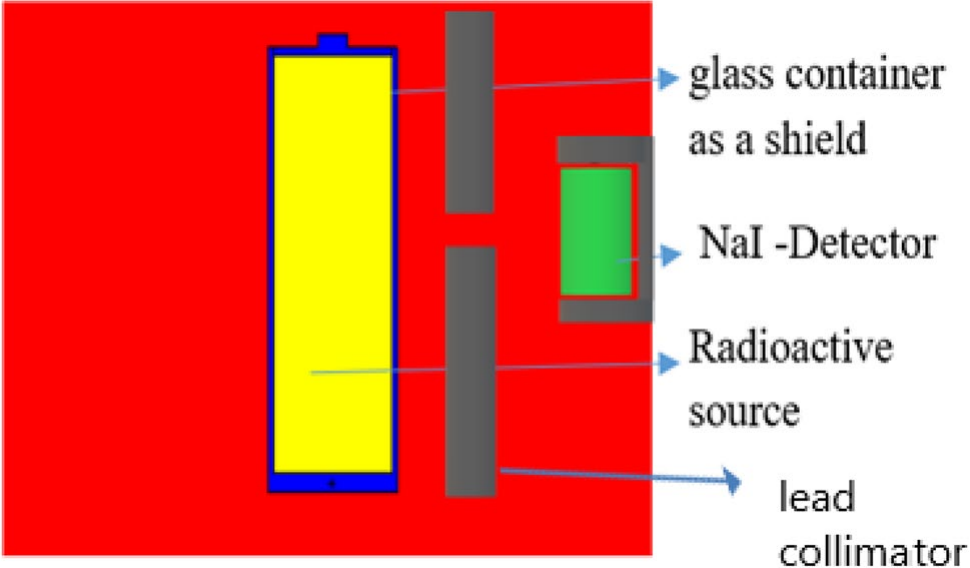
Geometry of simulated MCNP5 model configuration.

The MCNP5 simulation model was used to calculate the attenuation coefficient of gamma rays and radiation exposure doses behind each glass sample, as described in Fig. 1. The gamma radiation source was placed inside the glass container in the presence of a lead collimator between the glass container or source and detector in order to estimate the worker’s exposure doses. For capturing MCNP5 simulation data, Tally F4, F4 m, F5, and F5 m were utilized. Simulations were performed with 10,000,000 histories, and all results simulated by the MCNP-5 code were reported with less than 0.1% error. Additionally, mass stopping power and projected range values were estimated for protons using the SRIM Monte Carlo simulation code and the ESTAR program to calculate mass stopping power and the continuous slowing down approximation range for electrons. Also, many shielding parameters have been examined by Monte Carlo simulation code MCNP5 and Phy-X/PSD software to estimate radiation exposure doses at various distances, dose transmission%, and the excess lifetime cancer risk% for radiation workers behind the glasses.

Phy-X/PSD is online photon shielding and dosimetry (PSD) software available at https://phy-x.net/PSD that has been developed for the calculation of parameters relevant to shielding and dosimetry. Moreover, another parameter relevant to shielding, i.e., the fast neutron removal cross section (∑_R_), can be calculated for a compound or a mixture using this software^22^.

A Monte Carlo simulation computer model named Stopping and Range of Ions in Materials, or SRIM (formerly TRIM), is frequently used to calculate electronic and nuclear stopping powers and simulate the inelastic and elastic energy transfers from an energetic incident ion to target atoms. The SIRM software utilizes the Bethe-Bloch formula, which describes the energy loss of charged particles as they traverse through matter. This formula takes into account various factors, such as the density and composition of the material, as well as the energy and charge of the incident particles. By inputting these parameters, SIRM can provide accurate predictions of the  material stopping power for protons with specific energies^23^.

The ESTAR program calculates stopping power, density effect parameters, radiation yield tables, and the CSDA range for electrons. The CSDA range is a very close approximation to the average path length traveled by a charged particle as it slows down to rest^24^.

The loss in proton or electron KE is generally characterized by the concept of stopping power, which is defined as the rate of energy loss by a charged particle per unit path length in an absorbing medium. The stopping power usually has units of MeV cm^2^/g when called the mass stopping power^23^ by selecting a material and entering the desired energies, or using the default energies. Energies are specified in MeV and must be in the range of 0.001 MeV to 10,000 MeV^25^.

## Results and discussion

### FTIR absorption spectra

FTIR spectroscopy is the most dependable technique for analyzing the structure of both amorphous and crystalline materials by identifying the several structural groups in the material's structure and explaining the location of various metal oxides and their interactions with adjacent ions through the glassy structure. So it can be used to detect the structural changes that could take place due to different effects, e.g., composition change, irradiation process, etc^14^. As known, the main structural building units of the borate network are the trigonal BO_3_ units, which are allied to form six-membered boroxol rings, and the tetrahedral BO_4_ groups. Hosting modifying ions in the glassy network, e.g., alkali ions, transition metal ions, and rare earth ions, causes the fracture of B-O-B bonds, producing more non-bridging oxygen (NBO), followed by the alteration of trigonal planar sp^2^ BO_3_ units into tetrahedral sp^3^ BO_4_ groups with penta, tetra, tri, and di borate groups. The type and concentration of the introduced modifier ions and their behaviors with surrounding metal ions control this process of forming NBOs. Lead borate glass has a complicated structure different from the traditional alkali borate glasses. Contrasting the traditional alkali oxides, the heavy metal lead oxide (PbO) works in a dual behavior in the glass network as both modifier and former^11^.

According to some authors^11^, the ratio (R) of PbO in the glass network controls its influence. They expected that PbO participates in the network initially as a modifier at 0 ≤ R ≤ 0.33 mol%, where each Pb^2+^ ion acts at double charge balance to deliver the positive charges required for forming two tetrahedral borate (BO_4_)^−^ units. At R = 0.33, the role of cations starts to modify to provide PbO as a former oxide. Pb^2+^ ions can participate through strong covalent bonds to form compacted structural groups, e.g., PbO_4_ and/or PbO_3_ pyramidal units. Pb^2+^ cations and their connected oxygens in the pyramidal units work to diminish the ionic charge balance availability, lessening the rate of forming the four coordinated borons^11^.

**Figure 2:**
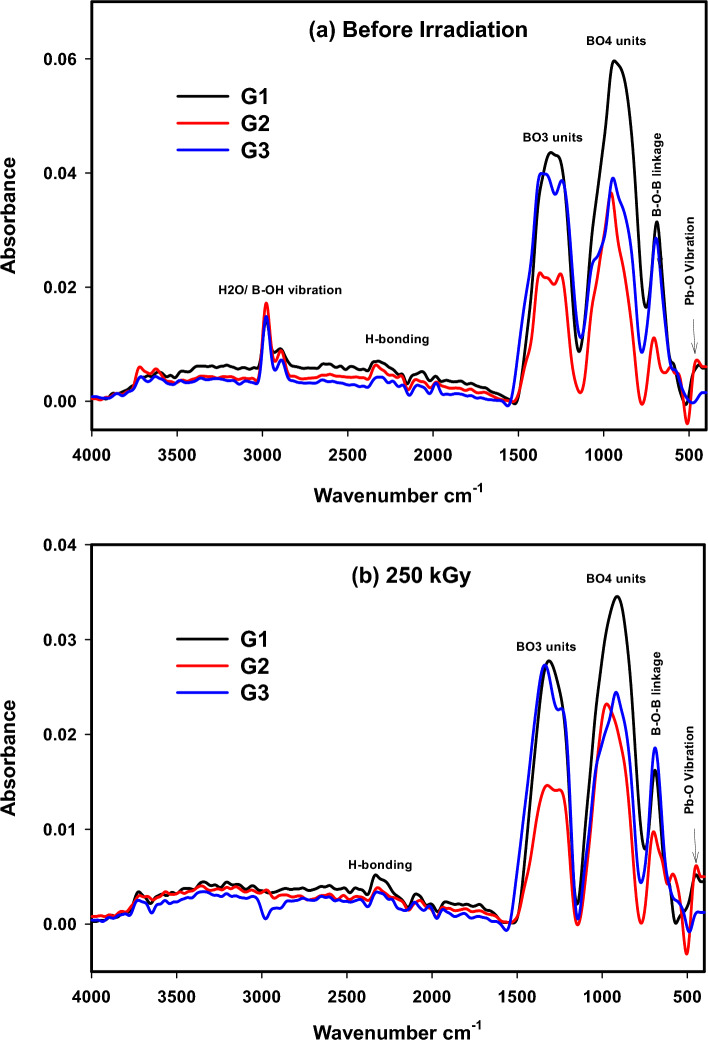
FTIR absorbance spectra of the prepared lead borate glasses before and after gamma irradiation with 250 kGy.

Figure 2 depicts the FTIR absorption spectra of the three considered lead borate glasses before and after gamma irradiation with 250 kGy. Comparing the spectra of the three prepared glasses, there are many common structural units, as follows:

1. A distinctive band in the far IR region (445–460–460 cm^−1^) at 445, 457, and 448 cm^-1^ for G1, G2, and G3, respectively, is ascribed to the vibration of building units of the glass network formed by heavy metal ions Pb^2+^^9,26^.
2. A slight broad band in the region from 570 to 610 at 587, 608, and 577 cm^−1^ correlated directly to the vibrational motions of Ce, Sb, and Mn cations in their network positions, respectively^14,15,27^.
3. A band centered at the medium area (~ 680–708 cm^−1^) at 687, 708, and 694 cm^−1^ is correlated to the bending vibrations of triangular B–O–B borate connections^28^.
4. A clear, sharp band at 940, 960, and 950 cm^−1^ for G1, G2, and G3, respectively, is correlated to the asymmetric stretching vibrations of B-O bonds in tetrahedral BO_4_ units^14^.
5. Quit broad bands at ~ 1200–1400 cm^−1^ assigned to stretching vibration of the trigonal BO_3_ units, such as the high peak at 1311 cm^−1^ for G1 and two high characteristic peaks at 1250–1380 cm^−1^ for G2 and 1370–1380 cm^−1^ for G3, attributed to the vibration of di-borate rings between B_3_O_6_-BO_3_ linkages and stretching vibration of tetraborate and/or penta-borate rings^14,29^.
6. Bands in the region 1600–4000 cm^−1^ e.g. broad bands around 2000 and 2300 cm^−1^ correlated to vibrations of H-bonding, and bands at 2890 and 2990 cm^-1^ correlated to the vibrations of molecular H_2_O, OH and B–OH units^14^.

Figure 2 also displays the FTIR absorption spectra of the three investigated glasses after being exposed to 250 kGy of gamma rays. The spectra reveal almost no main changes in the situation or intensity of the detected vibrational bands, indicating the large stability of lead-borate structures and the strong compacted networks. The closed and joined structures are supported by the highly polarizable heavy lead ions (Pb^2+^) that can block the path of gamma photons through the network and the effective role of the induced different metal ions. So outstanding stability and radiation shielding behavior against ionizing gamma radiation are observed.

#### Comparative analysis of deconvoluted FTIR spectra

**Figure 3:**
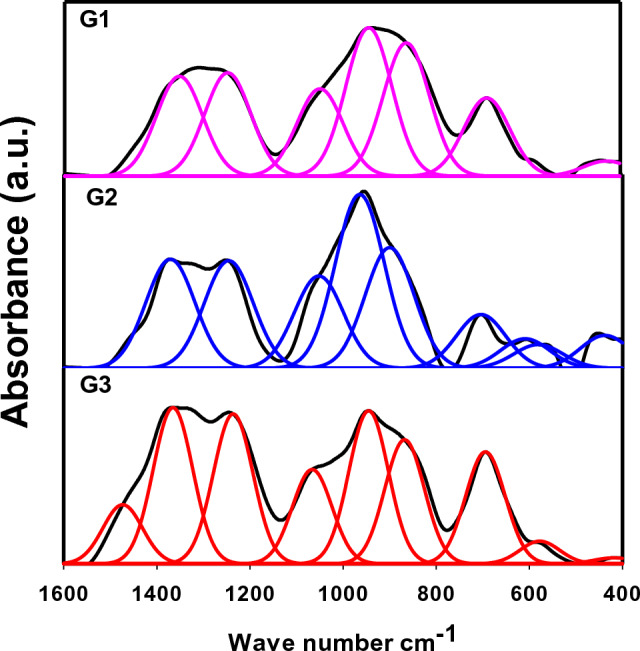
Deconvoluted FTIR absorbance spectra of the prepared lead borate glasses before irradiation (Gaussian Model).

**Figure 4:**
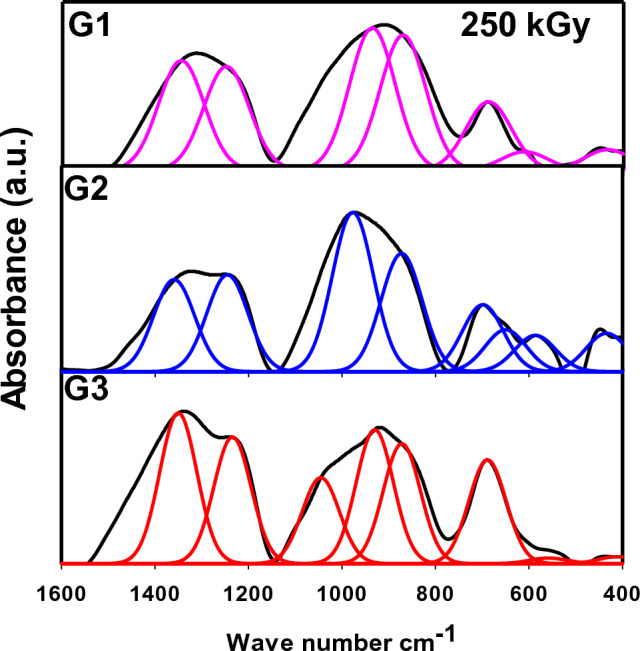
Deconvoluted spectra of the three prepared lead borate glasses after gamma irradiation with 250 kGy (Gaussian Model).

More details regarding the effect of the introduced metal ions and the influence of irradiation on the fabricated glasses can be interpreted simultaneously according to each glass composition in a comparative analysis using the deconvoluted spectra of the Gaussian model as shown in Figs. 3 and 4. The spectra of the fresh glasses before irradiation are displayed in Fig. 3. G1 (Ce glass) shows seven deconvoluted peaks at 440, 690, 865, 940, 1050, 1248, and 1353 cm^−1^. G2 (Sb glass) shows nine deconvoluted peaks at ~ 438, 580, 610, 702, 902, 966, 1054, 1250, and 1375 cm^−1^, while G3 (Mn glass) shows nine peaks at ~ 430, 577, 700, 868, 947, 1066, 1237, 1366, and 1480 cm^−1^. Assignments of the observed bands can be understood according to the following arguments:

- Bands at 430, 435, 438, 440 and 473 cm^−1^ can be recognized as the vibration of intermediated heavy metal Pb^2+^ ions as essential building units in their interstitial sites as PbO_4_/PbO_3_ units^9,18,27^. In addition to the vibrational motions of alkali modifier cations in their residing sites (bridging or non-bridging), e.g., Na^+^ or Al^3+^ ions^26^, the vibration of double degenerate bending vibrations of the SbO_3_ structural unit in G2^20^ and/or vibrational motions of the transition metal Mn^2+^ ions in their octahedral sites in G3^26^.
- Bands at 560 and 577, ~ 580–585 and 610, 650, 690 cm^−1^ ascribed to bending vibration of tetrahedral BO_4_ groups associated with modifier ions, e.g. Pb^2+^, Sb^3+^, K^+^, Na^+^, Al^3+^, and/or Mn^2+^/ Mn^3+^. Furthermore, the overlaid stretching vibration of Ce–O bonds in Ce^3+^/Ce^4+^ and ions in the four-fold coordination^19^ or asymmetric bending vibrations in the trigonal pyramids of SbO_3_^28^.
- Bands at ~ 689, 700, 702–704 cm^−1^ correlated to bridging oxygen vibration in BO_3_ units and bending vibration of B-O-B linkages, where oxygen is allied between BO_3_ and BO_4_ units^26^ e.g. bending vibrations of B-O-B linkages and B-O-Sb linkages in G2^28^.
- Bands at 865, 868, 870 and 873 cm^−1^ attributed to the triangle and tetragonal vibrations of Pb^2+^ ions and vibration of Pb–O-B linkages^9^. Furthermore, vibration of boroxol rings in G1, where the cerium ion can be associated with hydroxyl OH^−^ groups and overlapped with B–O–B links of the main tetrahedral BO_4_ borate structure, produces a noticeable bending vibration band at ~ 800 cm^−1^^14,29^.
- Main bands at 931, 938, 940, 947, 966- 977, 1066, 1048, 1237, 1233, 1366, and 1350 cm^-1^ correlated to the asymmetric stretching vibration of the B–O bonds in BO_4_ units and antisymmetric stretching vibrations of B–O–B vibrations for various borate units; tri, penta, and di-borate groups or B–O–B linkages^30^ e.g. triangle and tetragonal vibrations of Pb–O–B or O–Sb links^9^.
- Bands at 1050 or 1054 cm^−1^ related to the antisymmetric stretching vibrations of B–O–B bond stretching vibrations for numerous borate units; tri, penta and di-borate groups, e.g., B–O–B linkages^30^ or B–O–Sb linkages^20^.
- Bands at 1237, 1233, 1246 and 1250 cm^-1^ attributed to the stretching modes of trigonal BO_3_, typically in boroxol rings.
- Bands at 1390, 1375, 1362, 1412 and 1480 cm^−1^ ascribed to vibration of B-O symmetric stretching within BO_2_O^−^ units and varied types of borate groups, as well as vibration approaches of boron-oxygen in BO_3_ triangular units^9^.

Figure 4 discloses the deconvoluted spectra of the three glasses after 250 kGy of gamma irradiation. The contrast between their spectra can be individually discussed as follows:

1. The cerium (Ce) ion belongs to rare earth ions, which have necessary properties endorsed to their surface electronic configuration through separating electrons in the 4f orbital and in the exterior electrons orbital 5s^2^ 5p^6^^14^. Ce ions participate in the glass structure either as Ce^4+^ or Ce^3+^ according to their concentration, host glass composition, and melting temperature of their glasses^14,31,32^. Cerium ions inhabit the glassy network in more than one valence state through an equilibrium redox reaction among tri and tetravalent states (Ce^3+^  ↔ Ce^4+^) to balance the negative charges in the neighboring tetrahedrons, but the absolute ratio of Ce^4+^ can often be improved. Some authors^14,19^ stated that cerium ions participate in the glass structure in the interstitial sites by way of imperfections and differentiated them as fall or cracked bonds.
  As is observed in G1, the obvious decline in the intensity afterward irradiation, specifically bands around 800 cm^−1^ designates more joining bonds and more stability in the glassy structure after irradiation. It is evident from Figs. 3 and 4 that the FTIR spectra of Ce lead borate glass before and after irradiation have similar vibrational manners with no changes in the position or intensity of bands, and the slight shift of the bands from 440, 865, 940 and 1353 cm^−1^ to 437, 864, 938 and 1350 cm^−1^, refers to more stability in the glass structure and provides the connection of the glassy network. This manner is also approved by the high stability of the band at 1250 cm^−1^ that indicates no alteration of di-borate rings into tetraborate or tri-borate rings, referring to the stability in the glass structure^19^. Additionally, the stable band at 690 cm^−1^ designates the absence of de-polymerization process by the insertion of Ce^3+^/Ce^4+^ ions, referring to the improvement of the glass structural stability where the central borate building units (BO_4_ and BO_3_) are kept constant in their positions. So, bond rearrangements and more relaxed, glassy network can be released. The relaxation process in the amorphous glass structure includes the liberation of the extra stored energy to give more stable, relaxed glass. So, an equilibrium between the two valence states of cerium ions can be expected, where no more NBO is obtained by irradiation. The presence of a high percentage of heavy Pb^2+^ (50 mol%) ions in G1 with their characteristics of high polarizability and the presence of cerium ions with their own behavior in captivating radiation photons and absorbing defect color centers (commercially, CeO_2_ is widely used as a decolorizing agent), yields an outstandingly stable glass structure of Ce-Pb-B_2_O_3_ with surprising gamma radiation shielding effect.
2. Sb_2_O_3_ is a fragile glass network oxide; however, it can readily form glass by adding network modifier ions such as Li^+^, Na^+^, K^+^….etc. Some glass scientists assumed that enhancing the radiation shielding efficiency of Sb-glass could be carried out by the incorporation of WO_3_ and/or PbO as well as alkali ions to improve the glass shielding ability, particularly with the high PbO content ranging from 10 to 40 mol%^33^. Therefore, a strong gamma shielding performance can be expected for the prepared Sb-glass with 35 mol% PbO content. Sb_2_O_3_ and PbO are considered to be effective glass network intermediate oxides. Sb_2_O_3_ can modify the borate network (Sb_2_O_3_–B_2_O_3_ system), creating BO_4_ units at a slight level whenever the rest of Sb_2_O_3_ can form its particular matrix essentially as SbO_3_ trigonal pyramids. However, in the case of PbO–B_2_O_3_ glasses, the four-coordinated borons BO_4_ can exceed 0.5 in ~ 50 mol% PbO, where the former PbO_4_/2 pyramidal units contribute as glass formers in the glassy network^28^. So, the increase in Sb_2_O_3_ content at the expense of PbO causes a rise in BO_3_ content and a decline in BO_4_ content. This behavior is owing to the greater affinity of PbO to transform BO_3_ units into BO_4_ units than Sb_2_O_3_ in borate glasses.
  After irradiation, G2 reveals peaks at ~ 435,585, 650, 704, 873, 977, 1246, 1362 and 1412 cm^−1^ as shown in Fig.4. According to the present composition of G2 glass listed in Table 1 (with 35 mol% PbO), a slight conversion of BO_3_ to BO_4_ groups would take place by irradiation, where only minor changes in absorption spectra can be observed, as shown by comparing Fig. 3 with Fig. 4. The deconvoluted bands are shifted lightly to longer wavenumbers, referring to the influence of gamma radiation in creating some atomic transpositions in the glassy structure or electronic deficiencies that include modifications in the valence states of lattice or impurity atoms, resulting in very slight deviations in the bond angles and/or bond lengths of the main structural borate groups^16^, thus a limited conversion between BO_3_ and BO_4_ units can be expected. Nevertheless, the stable performance of the central vibrational bands in the fabricated Sb-PbO-B_2_O_3_ glass system against such high dose of gamma radiation (250 kGy) reflects its particular shielding behavior as a result of the weighty massive polarizable Pb^2+^ and Sb^3+^ ions that deferment the allowed passage of liberated electrons from irradiation, inhibiting then the formation of more induced weaknesses, leaving the main structural building groups almost persistent^14,15^.
3. Mn^2+^ as transition metal ions like Ti^4+^, V^5+^, Cr^3+^and Fe^3+^ ions are able to vary their valence states in the glassy structure reliant to their concentration and the main structure of the host glass. Mn^2+^ ion occupies the network as distorted octahedral or tetrahedral Mn^3+^ and can contribute in the glassy network in many coordination: Mn^2+^, Mn^3+^, Mn^4+^, MnO_4_^-^ and MnO_4_^2-^ ions or a combination of them^34^. Mn ion participates in the glassy structure either as network modifier or network former according to its concentration in the glass structure and the possible redox equilibrium of Mn^2+^/Mn^3+^ in alkali lead borate glass system^34^. At lower deliberations of MnO_2_, most manganese ions are in the form of the Mn^2+^ state with five unpaired electrons in their valence shell dispersed in t_2_g and eg orbitals. The octahedral Mn^2+^ ions can control the electronegativity decrease of the present modifiers, Na^+^ and Al^3+^ ions, corresponding to the increase in basicity of the surrounding anions in alkali borate glasses^34^. Mn^2+^ ions are differently bonded in the form of higher octahedral coordination; however, a small fraction of the lower tetrahedral coordination Mn^3+^ may be also found at higher Mn levels^35^. According to the last assumption, it is expected that manganese ions participate mainly in their tetrahedral coordination’s as Mn^3+^ ions, due to their contribution in a relatively high percentages (5 mol. %), in addition to some octahedral coordination as Mn^2+^.

After the irradiation process, G3 reveals a quite shift to lower wavenumbers to 430, 560, 689, 873, 931, 1048, 1233 and 1350 cm^-1^. By comparing the detected vibrational bands in Figs. 3 and 4 for G3 before and after irradiation, an obvious stability in the glass structure can be noticed, where the main vibrational bands are kept in their sites with a slight shift to lower wavenumbers, referring to saturation and/or relaxation in the glass structure. Some factors provide the stability of such structure and its resistance to irradiation, e.g., the highly polarizable Pb ions (PbO_3_/PbO_4_), the compacted AlO_4_ groups that strengthen the glass firmness, and essentially the occurrence of Mn^2+^/Mn^3+^ ions as transition metal ions having the ability to alter their valence states and absorbing the predicted defect centers caused via irradiation (-ve electrons/+ve holes). Thus, the highly compacted and rigid structure of G3 is observed, providing the glass with the ability to shield gamma radiation at such high dose (250 kGy).

**Figure 5:**
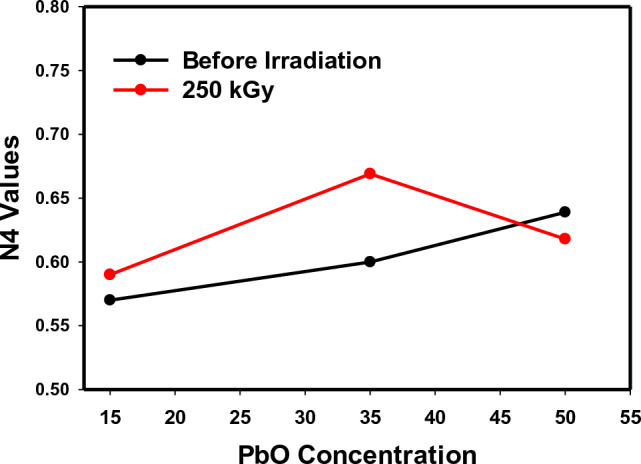
Variation of N4 values as a function of PbO content before and after gamma irradiation.

Fig. 5 displays the N4 values for each glass composition before and after gamma irradiation. N4 is a significant parameter that refers to a part of four coordinated boron atoms in the glass, determining the ratio of (BO_4_) groups and the possible conversion between the two main structural units of borate (BO_3_ and BO_4_). Thus, the glass's response to many external factors can be interpreted, such as variations in metal oxide content, the introduction of certain ions, heat treatment, irradiation process, etc. As obvious from Fig. 5, N4, for G1 with the higher PbO content (50 mol%) is decreased after irradiation, referring to more stable structure produced after the irradiation process. According to many authors, N4 is increased with PbO content up to 50 mol%, then further growth of PbO causes a drop in N4 value (ratio of BO_4_/BO_3_)^11^. This behavior approves the existence of more stable and relaxed structure of G1 glass (Ce–PbO–B_2_O_3_) without more NBO after irradiation. On the other hand, G2 and G3 obtain a slight increase in N4 values after irradiation, while the tendency of G3 (Mn–PbO–B_2_O_3_) to maintain a stable structure with lower N4 is greater than that of G2 (Sb–PbO–B_2_O_3_) because of the distinct impact of manganese ions in captivating radiation defect centers through changing their outermost configurations.

### Density

**Figure 6:**
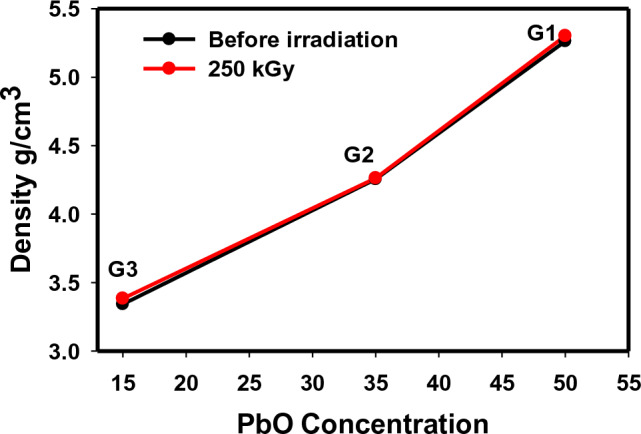
Density of the prepared lead borate glasses before and after gamma irradiation.

Figure 6 displays the density of the three studied glasses before and after exposure to gamma radiation at a dose of 250 kGy. The density of glass is a very important physical factor used to estimate the glass's firmness and rigidity. It describes a race between masses and volumes of the glassy units and in what way influentially the ions packed in the glassy structure^18^. As shown from Fig. 6, the three prepared glasses have density values of 5.26, 4.25, and 3.34 g/cm^3^ for 50, 35, and 15 PbO-containing glasses, G1, G2, and G3, respectively. The values indcate a direct correlation with the concentration of heavy lead ions in each glass composition because density can be well-defined mainly as weight per unit volume, so it is directly interrelated to the molecular weights of the induced ions in the material structure. Therefore, a rise in density values is observed with Pb addition due to the replacement of lower atomic masses in the glass composition with the higher atomic mass of PbO (Pb^207.2^)^18^. After the irradiation process, the density of the glass reveals approximately the same values as those before irradiation, with slightly higher values after irradiation of 5.30, 4.26, and 3.38 for G1, G2, and G3, respectively. This observation indicates the positive effect of ionizing radiation, where more compactness in the glass network can be obtained, giving narrower interstices and more closed systems against the  high dose of gamma radiation.

### Electron spin resonance ESR

**Figure 7:**
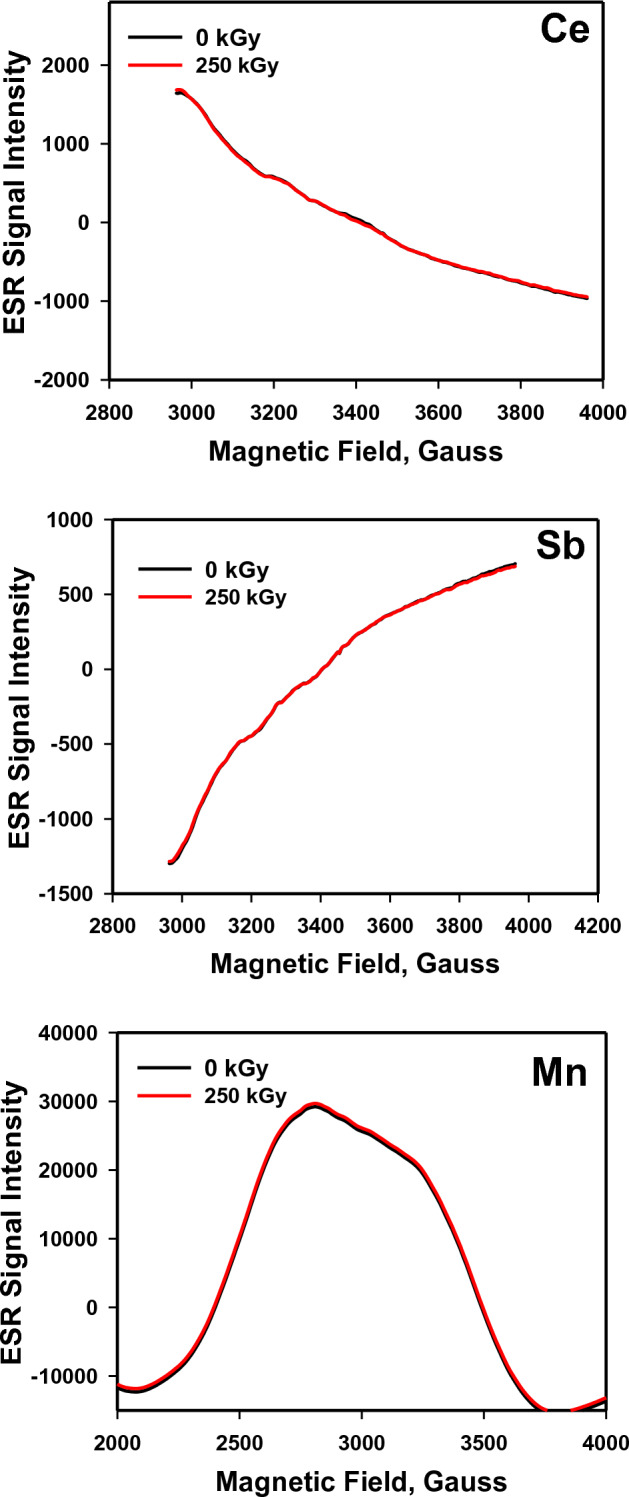
ESR spectra of the prepared lead borate glasses before and after irradiation.

The non-destructive technique ESR is used for distinguishing the unpaired electrons made by exposing materials to external effects such as ionizing radiation. Intensity and signals on the ESR spectrum depend predominantly on the number of free radicals formed by irradiation influence, e.g., positive holes and/or negative electrons at diverse trapping positions on the glass structure. Figure 7 displays the impact of 250 kGy of gamma radiation on the ESR spectra of the three investigated lead borate glasses, and the spectra demonstrate that the spectra appear very identical for each glass before and after the irradiation process, in addition to the complete absence of real sharp ESR signals in the three spectra either before or after exposure to gamma rays. The asymmetry of the broadening features obtained between the three spectra can be attributed to the dissimilar compositions of the glasses and the different contributions of the induced ions in various valence states through the exchange coupling in the glass clusters^19^. The obvious, non-distinctive changes in the spectra positions and intensity for the three glasses after 250 kGy of gamma radiation indicate largely the insignificant impact of irradiation on the glass structures, where there are no free radicals formed by irradiation. Accordingly, the high stable structure or the resistance effect of the prepared glasses to ionizing irradiation at such high dose. The highly polarizable Pb^2+^ ions and the precious effect of each induced ion, Ce, Sb, and Mn, are the main reasons for forming the blocked glass system that can obstruct the way of radiation photons until they miss their energies through molecules, leaving the main structure without deviations and verifying the high tendency of the glasses to dodge or shield high doses of gamma radiation.

### Shielding parameters

**Figure 8:**
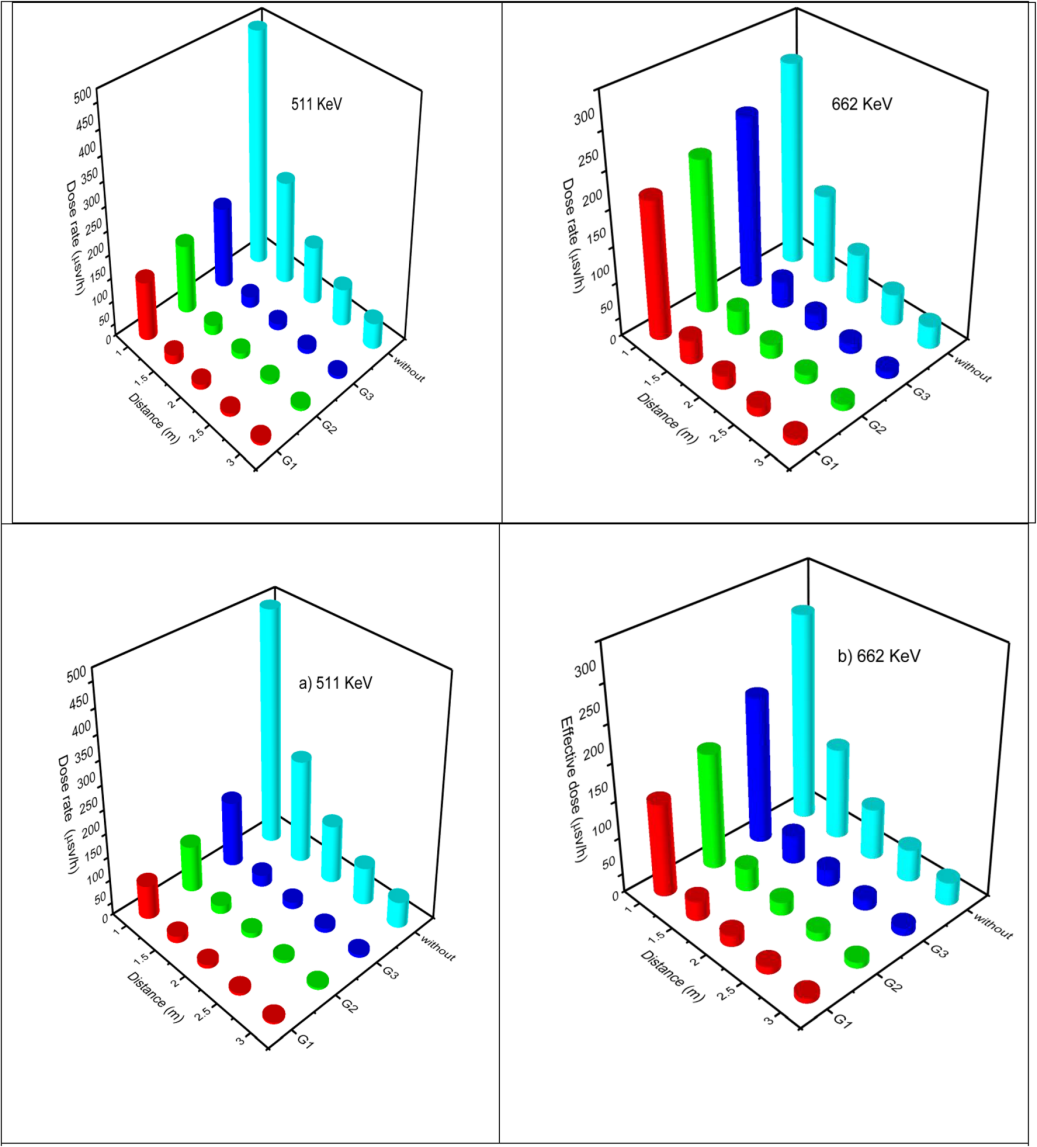
(**a**,**b**) Variation of simulated workers gamma doses rate (µSv/h) from exposure to radioactive source (at E = 511 keV and E = 662 keV) at different distances (m) of the prepared glass containers with 1 cm (**a**) and 2 cm (**b**) thicknesses.

Figure 8 reveals the simulated dose rate (mSv/h) for each glass sample with thicknesses of 1 cm and 2 cm glass containers at distances (D) of 1 m, 1.5 m, 2 m, 2.5 m, and 3 m compared to the shield (N.S.) against exposure to radioactive sources (with photon energy of 511 keV (F-18) and 662 keV (Cs-137) and activity of 100 mCi, where the dose rate decreases with increasing either the distance or the thickness of the glass shield containers.

As obviously revealed from Fig. 8, the shielding efficiency order of the simulated glass containers is G3 < G2 < G1 depending on the order of decrease in dose rate (mSv/h). Furthermore, consistent with the ALARA concept of radiation shielding, the distance away from the source and thickness of the shield are very important factors to be detected. The results also show that the maximum dose rates appear at a distance of 1 m with a glass container of 1 cm thickness. While the minimum dose rates appear at a distance of 3 m with a glass container of 2 cm thickness, Additionally, the energy of the radioactive source plays a main role in determining the exposure dose rate. The dose rate from a radioactive source with a photon energy of 511 keV is less than that with a photon energy of 662 keV at the same distance and glass thickness, as shown in Fig. 8. It is noticeably that the dose rates of gamma radiation decrease as the density of the glassy containers rises as a result of the rise in absorption coefficient that leads to the absorption of a large proportion of photons, reducing the radiation source intensity. Also, the portion of the scattered photons would increase with increasing energy.

The rate at which the investigated glasses transmit gamma doses can be detected by the dose transmission percentage, which is another important shielding parameter used for detecting the fraction of the shielded gamma dose to the unshielded gamma dose^36^ according to the following equation:

2 $$\mathrm{Dose \,Transmission\, \% }= \frac{\mathrm{ dose\, with\, shield }}{\mathrm{ dose \,without\, shield}}\times 100\%$$

**Figure 9:**
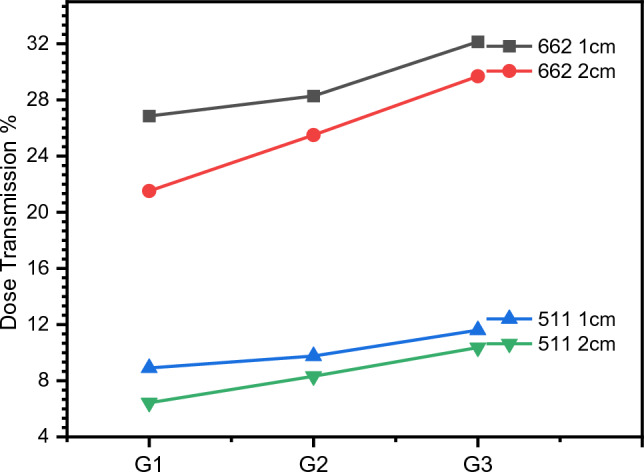
Dose transmission % of radioactive sources (511 keV and 662 keV) in 2 cm thickness glass containers at a distance 2m.

By using the simulated MNCP5, the dose transmission% for each glass shield with 1 and 2 cm thickness and a distance of 2 m away from 511 and 662 keV energies is determined and epitomized in Fig. 9, where the dose transmission% decreases with growing PbO mol%. So, it appears smaller for G1 (with the highest PbO mol%) than G2 and G3 (with lower PbO mol%), indicating that G1 is a better shielding material for gamma sources than G2 and G3.

At gamma energy 662 keV, the dose transmission% reduces from 26.9 to 21.5% by increasing the thickness of G1 from 1 to 2 cm, while at 511 keV it decreases from 8.9 to 6.4%. Likewise, G2 decreases from 28.3 to 25.5% at 662 keV and from 9.7% to 8.3% at 511 keV, and G3 decreases from 32.1 to 29.7% at 662 keV and from 11.6 to 10.4% at 511 keV.

In order to reflect the potential health danger of radiation, one of the most vital studied parameters is the excess lifetime cancer risk % which evaluates the glass shields efficiency according to the point of view of health hazards. It can also differentiate between glass shields and other working shields in the radiological and nuclear fields.

The excess lifetime cancer risk due to gamma radiation, based on the effective dose using the simulated MCNP5 code values, is determined as follows^37^.

Excess lifetime cancer risk = Effective dose Mean duration of life Fatal cancer risk factor.

where mean duration of life and fatal cancer risk factor are the life duration (40 years) and fatal cancer risk factor for stochastic effect (0.04 Sv^-1^ for the workers) were used by the ICRP (international Commission on Radiological Protection) for stochastic effects, respectively^37^.

**Figure 10:**
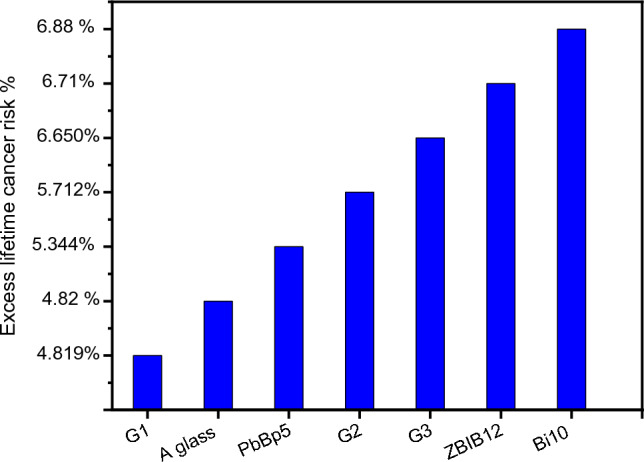
Comparison between excess lifetime cancer risk % values for workers due to the exposure to radioactive source (E = 662 keV) inside the three studied glasses containers (2 cm thickness) and other glasses shields at 2m distance.

Figure 10 displays obviously the excess lifetime cancer risk % for workers with 2000 h per year for 40 years working^38^ from radiation exposure dose at 2 m far away the investigated glass shields and compares our studied samples to some of the glass shields from earlier study publications by employing the weight percentages of their elemental compositions in our simulated MCNP5 code to calculate the effective doses behind these glasses under identical conditions: A-glass^ 39^, PbBaP5^40^, ZBiB^41^ and Bi10^42^. The results reveal that G1 glass shield diminishes the lifetime cancer risk better than the other formerly studied shields, while G2 and G3 lessen the lifetime cancer risk better than ZBiB^41^, and Bi10^42^ biological shields. Lifetime cancer risk % indicates the effectual behavior of the studied glass shields in diminishing the lifetime cancer risks and their high importance in avoiding workers biological radiation dangers.

Recent research has shown that charged particles have radiobiological effects and result in defects in shield structures due to photon effects. Mass stopping power is a very important parameter used to ensure the security of radiation workers, who must have an adequate attenuator. Therefore, it's crucial to distinguish the mass-stopping power of the glass shields and their ranges in the shield.

An energetic ion misses energy nuclear reactions and bremsstrahlung, elastic energy transmission to atomic nuclei, and inelastic energy transmission to electrons (excitation and ionization of the target atoms and the ion itself). Stopping power, commonly identified as stopping force, is a unit of measurement for the rate of energy loss per unit route length (dE/dx). Ion energy loss can be divided into two categories: (1) energy transfer to target electrons, which causes ionization, and (2) energy transmission to target nuclei, which causes atomic dislocations or phonon energy debauchery for energy transmission overhead or under the threshold movement energy^43^.

In this part, it is recommended to examine how the charged particles (electrons) move through the materials by using the Esther code, which is based on Monte Carlo simulation. In reality, it can be noticed that the effective radiation quantity in a material, changes in response to the change in energy value, as per ψ_e_ calculations in (MeVcm^2^/g). The ψ_e_ results can be used to detect the material's radiation shielding capacity^44^. The software tool that accounts for ψ_e_ of the examined glasses for electron particles (Estar), refers to the decrease in electron kinetic energy as it travels through the glass samples with a certain density so ψ_e_ is regarded as a strong sheild factor.

**Figure 11:**
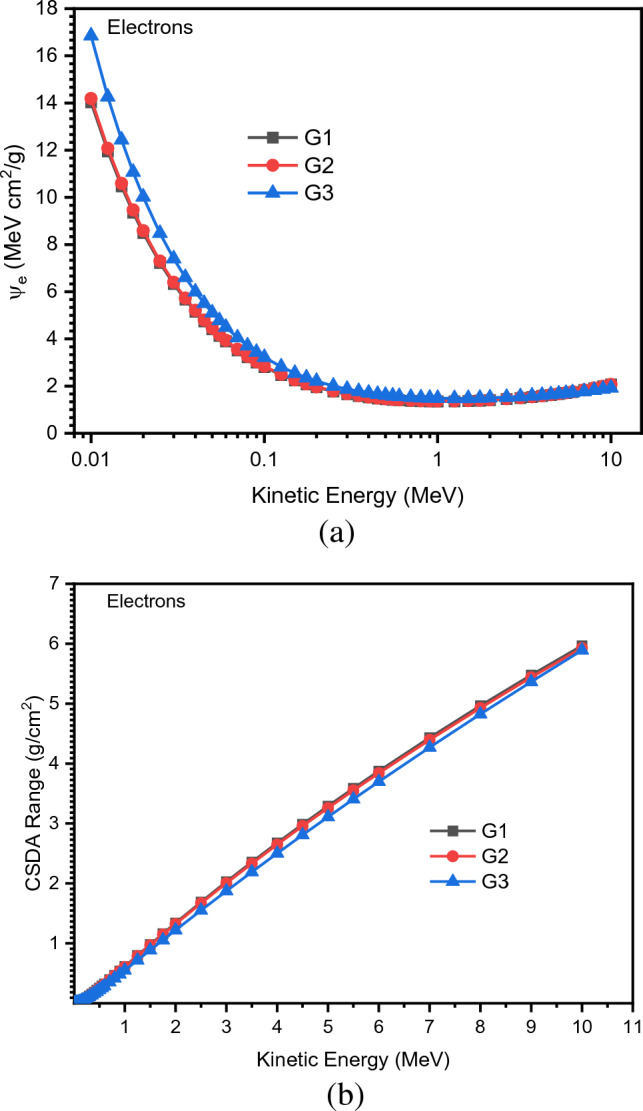
(**a**) Variations of electrons mass stopping power (ψe) with different kinetic energy of electrons. (**b**) Variations of CSDA range with different kinetic energy of electrons.

For the samples under examination, Fig. 11(a, b) shows the fluctuation of ψ_e_ and CSDA values as a function of kinetic energy (0.010–10 MeV). It is observed that the, CSDA range increases with increasing kinetic energy, while ψ_e_ values decrease with energy. Moreover, the lowest ψ_e_ and largest CSDA range values are found for the highest Pb concentration (G1 glass). The CSDA lengthens to express the typical range of stopping any charged particles with kinetic energy (KE), and its values are relational to kinetic energy and rise very slightly with increasing Pb concentrations. This is because charged particles can penetrate the glass network more deeply where the interaction cross-sectional region of the particles decreases with the increase in KE.

**Figure 12:**
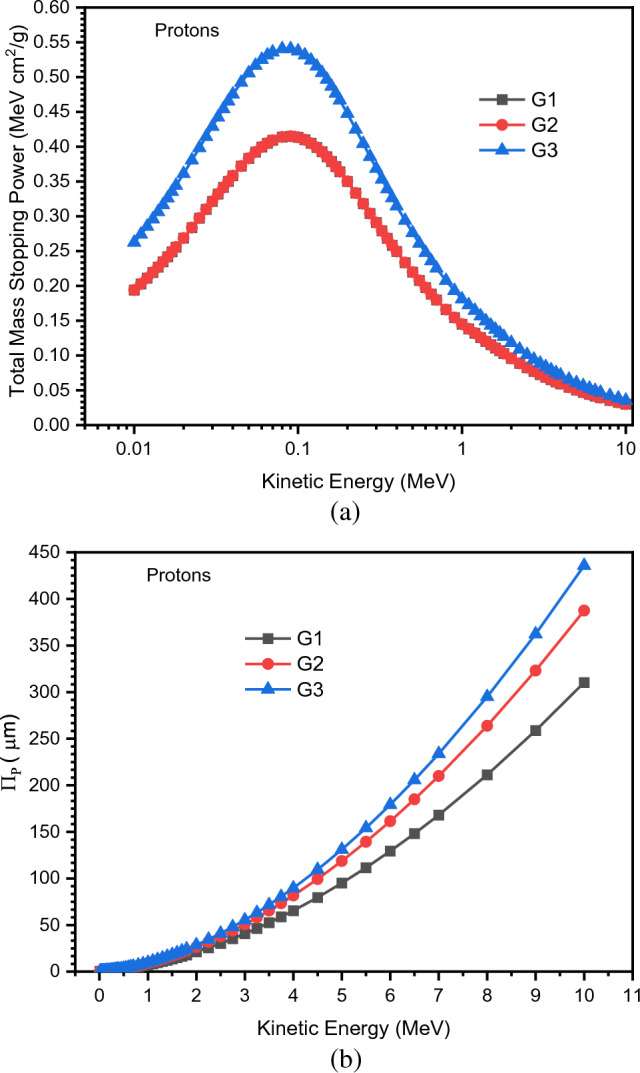
(**a**) Variations of proton mass stopping power (ψ_p_) with different kinetic energy of electrons. (**b**) Variations of projection range (Π_P_) of proton with different kinetic energy of electrons.

Figure 12a,b shows how the proton mass stopping power (ψ_p_) and projection range (Π_P_) change with KE, where the greatest proton-protective material needs the least Π_P_. As shown in Fig. 12b, G1 glass delivers complete proton protection as it has the lowest Π_P_ value compared to G2 and G3 samples.

Because proton particles have a higher mass (1.67262 × 10^−27^ kg) than electrons (9.1093837015 × 10^−31^ kg), which is only 1/1,836 the mass of the proton, they have a lower velocity, allowing protons to have smaller values of ψ_p_ than electrons ψ_e_ at the same energy.

The macroscopic effective range cross-section of the fast neutron, ∑_R_, is used to quantify the chance of a fission neutron to react in a medium that eliminates it from a neutron beam of fission energy (by decelerating through elastic scattering). It is desirable to use light and heavy elements in shield composites for optimal fast neutron attenuation.

**Figure 13:**
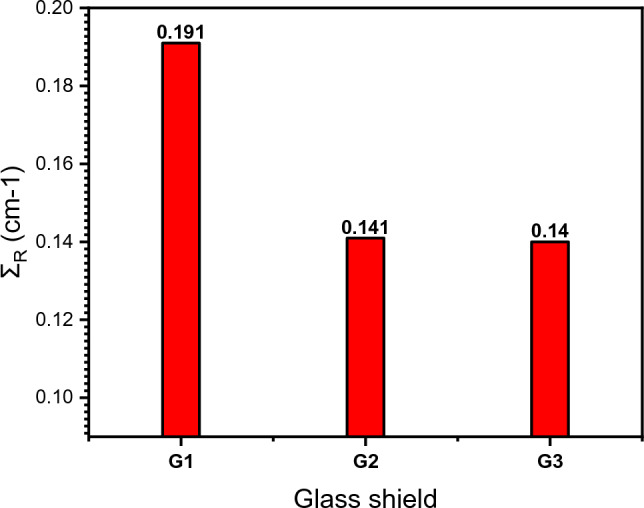
Macroscopic effective removal cross-section for fast neutron of the studied glasses.

∑_R_ changes with the energy of fast (fission) neutrons, is thought to remain persistent for energies between 2 and 12 MeV for neutrons. Figure 13 displays the ∑_R_ (cm^-1^) values of the examined glasses by Phy-X/PSD software. The graph demonstrates how ∑_R_ steadily declines as PbO content decrease, where the sample G1 has a much higher Ʃ_R_ than G2 and G3. This indicates a nominal improvement in the neutron-shielding performance for G2 and G3 than G1, which is a good behavior compared with some typical shielding materials, e.g. water = 0.102 cm^−1^, graphite = 0.077 cm^−1^, ordinary concrete = 0.094 cm^−1^ and hematite-serpentine concrete = 0.097 cm^−1^^45^.

G1 glass sample compares favorably to these materials when the value of (Ʃ_R_ cm^-1^) is exceeded, demonstrating that it is a more effective fast neutron attenuator.

## Conclusion

The FTIR spectra of the three investigated Ce, Sb or Mn lead borate glasses reveal high structural steadiness against 250 kGy of gamma radiation. The spectra of Ce-glass display the same vibrational bands without changes in their position or intensity after irradiation, and the slight shift of the bands from 440, 865, 940 and 1353 cm^−1^ to 437, 864, 938 and 1350 cm^−1^, refers to stability and connectivity of the glass structure, since the stability of the band at 1250 cm^−1^ indicates no transformation of di-borate rings into tetraborate or tri-borate rings. The spectra of Sb-glass also reveal a stable performance after gamma irradiation thanks to the heavy, massively polarizable Pb^2+^ and Sb^3+^ ions that defer the free passage of radiation photons, leaving the structural building units almost constant. Mn ions containing glass reveal also a stable structure correlated to their nature as transition metal ions able to adjust their valence states (Mn^2+^/Mn^3+^) to captivate radiation defect centers (− ve electrons/ + ve holes), in addition to the compacted AlO_4_ groups that strengthen the glass rigidity. Attendance of the greatly polarizable Pb^2+^ ions in the three fabricated glasses is the main reason for such structural stability by forming strong covalent bonds, e.g., PbO_4_ and/or PbO_3_ pyramidal units that work to lessen the ionic charge balance accessibility. Accordingly, the higher the Pb content, the more stable and shielded the glass because PbO contributes to a dual behavior as a glass former and modifier, enhancing the glass structure's solidity. The decrease in N4 values after irradiation for Ce-glass with the higher PbO content (50 mol. % PbO), confirms its higher structural stability and relaxation than Sb-glass (35 mol. % PbO) and Mn-glass (15 mol. % PbO) with lower PbO content. The density of the glasses shows a direct relationship with the heavy PbO content, as it depends mainly on the molecular weights of the induced ions. So the replacement of lighter atomic masses in each glass composition by the heavier atomic mass of PbO (Pb^207.2^) causes a higher density for Ce glass than Sb and Mn glasses.

After irradiation, the values of density reveal a slight increase, which designates the positive influence of ionizing radiation on the glass compactness by narrowing the glassy network interstices, giving more closed systems. ESR spectra exhibit very identical behavior before and after 250 kGy of gamma radiation, and the complete absence of real sharp ESR signals indicates the absence of free radicals formed by irradiation, and the high resistance of the glass structure to such high gamma rays. Many radiation shielding parameters were evaluated, e.g., mass stopping power and projected range values for proton particles, using the SRIM Monte Carlo simulation code and the ESTAR program to calculate mass stopping power and the progressive slowing down approximation range for electrons.

For the three glasses, G1, G2, and G3, the maximum values of ψp are 0.415, 0.415 and 0.541 MeV cm^2^/g at energy of 0.09 MeV, respectively. While ψe values at 10 MeV are 2.073, 2.069 and 1.92 MeV cm^2^/g, and the electron CSDA range upsurges with electron KE, at KEs of 10 MeV, the CSDA values are 5.968, 5.930 and 5.892 g/cm^2^ and Π_P_ for proton 310.1, 387.5 and 435.8 μm for G1, G2, and G3 samples, respectively. The simulation of radiation dose rates (511 keV or 662 keV) at different distances for each glass (1 and 2 cm thickness) by MCNP5 code and Phy-X/PSD software follows the order of shielding efficiency as (Ce-glass) G3 < (Sb-glass) G2 < (Mn-glass) G1. The dose rate% decreases as the density of the glass containers increases due to the increase in absorption coefficient and thus the absorption of large proportions of photons, reducing the intensity of the radiation source and giving the best behavior at a distance of 2 m in the 2 cm thickness of the glass container. On the other side, the dose transmission % decreases with increasing PbO mol. %, so it appears smaller for G1 (with the highest PbO mol. %) than G2 and G3. Comparing the excess lifetime cancer risk % for workers (working 2000 h per year for 40 years due to radiation exposure at 2 m away from the glass containers of a radioactive source) with other shielded materials, reveal the ability of Ce- glass shield (G1) to reduce lifetime cancer risk better than many other shields. Additionally, the lowest ψ_e_ and largest CSDA range values are found for the highest Pb concentration (G1 glass). Macroscopic effective range cross-section of the fast neutron ∑_R_ (cm^−1^) from Phy-X/PSD software displays a steady increase with PbO content, giving the order of increase: G3 < G2 < G1.

The closed and associated structures are supported by the highly polarizable heavy lead ions (Pb^2+^) that can block the path of gamma photons through the network and the effective role of the induced different metal ions. So outstanding stability and radiation shielding behavior against high ionizing radiation up to 250 kGy are obtained, specifically for the glass with the maximum PbO content G1 (Ce-glass). The whole results recommend the promising usage of the prepared glasses as storage containers for radioactive wastes, especially for nuclear medicine entities in hospitals or other amenities.

## Data Availability

The datasets used and/or analyzed during the current study available from the corresponding author on reasonable request.
